# New insights into the regulation of plant metabolism by *O*-acetylserine: sulfate and beyond

**DOI:** 10.1093/jxb/erad124

**Published:** 2023-04-07

**Authors:** Anastasia Apodiakou, Rainer Hoefgen

**Affiliations:** Max Planck Institute of Molecular Plant Physiology, Am Mühlenberg 1, D-14476 Potsdam-Golm, Germany; Max Planck Institute of Molecular Plant Physiology, Am Mühlenberg 1, D-14476 Potsdam-Golm, Germany; Hasselt University Belgium

**Keywords:** APR, ATTED-II, BGLU28, gene regulation, network, *O*-acetylserine, oxidative stress, ROS, SDIs, sulfur, sulfur deficiency, SLIM1

## Abstract

Under conditions of sulfur deprivation, *O*-acetylserine (OAS) accumulates, which leads to the induction of a common set of six genes, called OAS cluster genes. These genes are induced not only under sulfur deprivation, but also under other conditions where OAS accumulates, such as shift to darkness and stress conditions leading to reactive oxygen species (ROS) or methyl-jasmonate accumulation. Using the OAS cluster genes as a query in ATTED-II, a co-expression network is derived stably spanning several hundred conditions. This allowed us not only to describe the downstream function of the OAS cluster genes but also to score for functions of the members of the co-regulated co-expression network and hence the effects of the OAS signal on the sulfate assimilation pathway and co-regulated pathways. Further, we summarized existing knowledge on the regulation of the OAS cluster and the co-expressed genes. We revealed that the known sulfate deprivation-related transcription factor EIL3/SLIM1 exhibits a prominent role, as most genes are subject to regulation by this transcription factor. The role of other transcription factors in response to OAS awaits further investigation.

## Teaser

*O*-Acetylserine (OAS), a sulfate deprivation marker, accumulates under stress conditions. We provide information about the function and regulation of OAS cluster genes and an OAS cluster co-expression network.

**Box 1. *O*-Acetylserine and cysteine biosynthesis**

*O*-Acetylserine (OAS) is synthesized from serine via serine acetyltransferase (SERAT) from the nitrogen and carbon assimilation pathway, providing the backbone yielding cysteine (Hoefgen and Nikiforova, 2008). In Arabidopsis five genes encoding SERAT proteins can be found: SERAT2;1 (AT1G55920) and SERAT2;2 (AT3G13110), which are localized in plastids and mitochondria, respectively, and SERAT1;1 (AT5G56760), SERAT3;1 (AT2G17640), and SERAT3;2 (AT4G35640), which are localized and expressed in the cytosol (Watanabe *et al.*, 2008; Krueger *et al.*, 2009). When plants experience reduced sulfate levels, OAS accumulates concomitant with the induction of *SERAT2;2* and *SERAT3;2*, and to a lesser extent, and mainly in roots, with that of *SERAT3;1* and *SERAT2;1* (Watanabe *et al.*, 2015; De Kok *et al.*, 2017; Watanabe and Hoefgen, 2019; Dietzen *et al.*, 2020). The mitochondrial SERAT2;2 is the enzyme that contributes most to OAS formation while having the highest activity (Watanabe *et al.*, 2008). It has been shown that SERAT and OAS thiol lyase (OASTL) can form a hetero-oligomeric cysteine synthase complex (CSC), which is stabilized by the presence of sulfide and can be dissociated by OAS availability (Hell and Wirtz, 2011). Thus, SERAT can affect and contribute to the control of cysteine synthesis (Hell and Wirtz, 2011; Maruyama-Nakashita, 2017). SERAT3;1 and SERAT3;2 are able to synthesize OAS independently of the CSC, presumably allowing the accumulation of OAS under conditions where the CSC is dissociated (Watanabe *et al.*, 2015). It has been shown that in the CSC, SERAT is activated, while OASTL is inactive, functioning as a regulatory subunit for SERAT. As a result, the produced OAS dissociates the complex and is further converted to cysteine by free OASTL (Feldman-Salit *et al.*, 2009). Cysteine feedback inhibits SERAT3;1 and SERAT3;2 activity, further supporting their specific function under sulfate deprivation as resupply of sulfate leads to synthesis of cysteine and a shutdown of the CSC-independent OAS production, thus preventing an overshoot of cysteine production (Watanabe *et al.*, 2015).

**Box 2. Sulfate assimilation and cysteine synthesis**

Sulfate (SO_4_^2–^) which has been taken up by sulfate transporters (SULTRs), is transported to the shoot via the xylem and eventually to the plastids. ATP sulfurylase (ATPS) forms 5-adenylylsulfate (APS) (Murillo and Leustek, 1995). APS provides a branchpoint and can follow different pathways. First, it can be reduced to sulfide following a two-step reaction which is catalyzed by APS reductase (APR) (Rotte and Leustek, 2000) to form sulfite and by sulfite reductase (SiR) to form sulfide (Rotte and Leustek, 2000; Saito, 2004; Hell and Wirtz, 2011; Takahashi *et al.*, 2011; Naumann *et al.*, 2018). Finally, sulfate will be incorporated into cysteine, the first organic form of the pathway. Sulfide and OAS are converted to cysteine by OASTL (Hell and Wirtz, 2011). Cysteine is further used as the backbone to form a huge number of sulfur-containing compounds such as glutathione (GSH), methionine, glucosinolates (GSLs), or further metabolites, vitamins, or lipids (Leustek *et al.*, 2000; Saito, 2004; Davidian and Kopriva, 2010; Takahashi *et al.*, 2011; Kopriva *et al.*, 2012). Alternatively, APS can be phosphorylated to form 3ʹ-phosphoadenosine-5ʹ-phosphosulfate (PAPS) by APS kinase (APK) being involved in sulfation reactions.

## Introduction

*O*-Acetylserine (OAS) has been shown to accumulate under sulfate-deprived growth conditions (Boxes 1, 2) and has been suggested to be a signaling molecule under these conditions due to its inverse correlation with sulfate content (Saito, 2004). External (Hirai *et al.*, 2003) or internal application (Hubberten *et al.*, 2012b) of OAS further supported this hypothesis. In addition to the accumulation of OAS under sulfate deprivation, further conditions could be identified that lead to the accumulation of OAS, even under sulfate-sufficient conditions (Espinoza *et al.*, 2010; Caldana *et al.*, 2011; Hubberten *et al.*, 2012b). A set of six genes was identified as being induced when OAS accumulates, termed OAS cluster genes (Box 3) (Hubberten *et al.*, 2012b). Conditions such as herbicide treatment with menadione were accompanied by the accumulation of reactive oxygen species (ROS) as a hallmark of stress. ROS accumulation leads to the induction in particular of serine acetyltranferase 2;1 (*SERAT2;1*) and somewhat less *SERAT2;2* (Watanabe *et al.*, 2015; De Kok *et al.*, 2017; Watanabe and Hoefgen, 2019), while under these conditions *SERAT3;2* and *SERAT3;1* are not induced. Treating Arabidopsis roots with menadione resulted in the accumulation of OAS after 0.5 h and showed a peak at 6 h of treatment (Lehmann *et al.*, 2009, 2012). OAS accumulation correlated to SERAT transcript accumulation and OASTL transcript reduction. Presumably, certain oxidative stress conditions in *Arabidopsis thaliana* result in OAS accumulation. Sulfur metabolism is activated in roots as a response to oxidative stress (Lehmann *et al.*, 2009). Further, it is speculated that jasmonate biosynthesis and the sulfur pathway interact (Jost *et al.*, 2005). The transcript profile of jasmonate-regulated genes is comparable with sulfur deprivation and OAS application experiments (Hirai *et al.*, 2003; Saito, 2004). *SERAT3* was one of the numerous genes that were induced under methyljasmonate (MeJa) treatment. OAS accumulation is thus a response ­facilitated through specific regulatory circuits employing different SERAT groups in order to synthesize OAS as a response to different stresses.

## The downstream function of OAS cluster genes

**Box 3. *O*-Acetylserine cluster gene abbreviations**

SDI1 SULFUR DEFICIENCY INDUCED 1

SDI2 SULFUR DEFICIENCY INDUCED 2

LSU1 RESPONSE TO LOW SULFUR 1

SHM7/MSA1 SERINE HYDROXYMETHYLTRANSFERASE 7/ MORE SULFUR ACCUMULATION1

ChaC/GGCT2;1 GAMMA-GLUTAMYL CYCLOTRANSFERASE 2;1

APR3 APS REDUCTASE 3

OAS accumulation induces the expression of the core OAS cluster genes (Box 3): *APR3* (AT4G21990), *SDI1* (AT5G48850), *SDI2* (AT1G04770), *LSU1* (AT3G49580), *SHM7/MSA1* (AT1G36370), and *ChaC/GGCT2;1* (AT5G26220) (Hubberten *et al.*, 2012b). To date, the functions of these genes have been partially resolved. Common to all of them is that they seem to be functionally involved not only in the sulfate deprivation response but also in other metabolic and physiological processes, probably having metabolic functions (*GGCT2;1*; *APR3*) or acting as upstream regulators (*SDI1*, *SDI2*, and *MSA1*), but also still other unclear functions such as being putatively involved in various processes as ethylene responses and autophagy (*LSU1*).

### SDI1 and SDI2

**Fig. 1.**
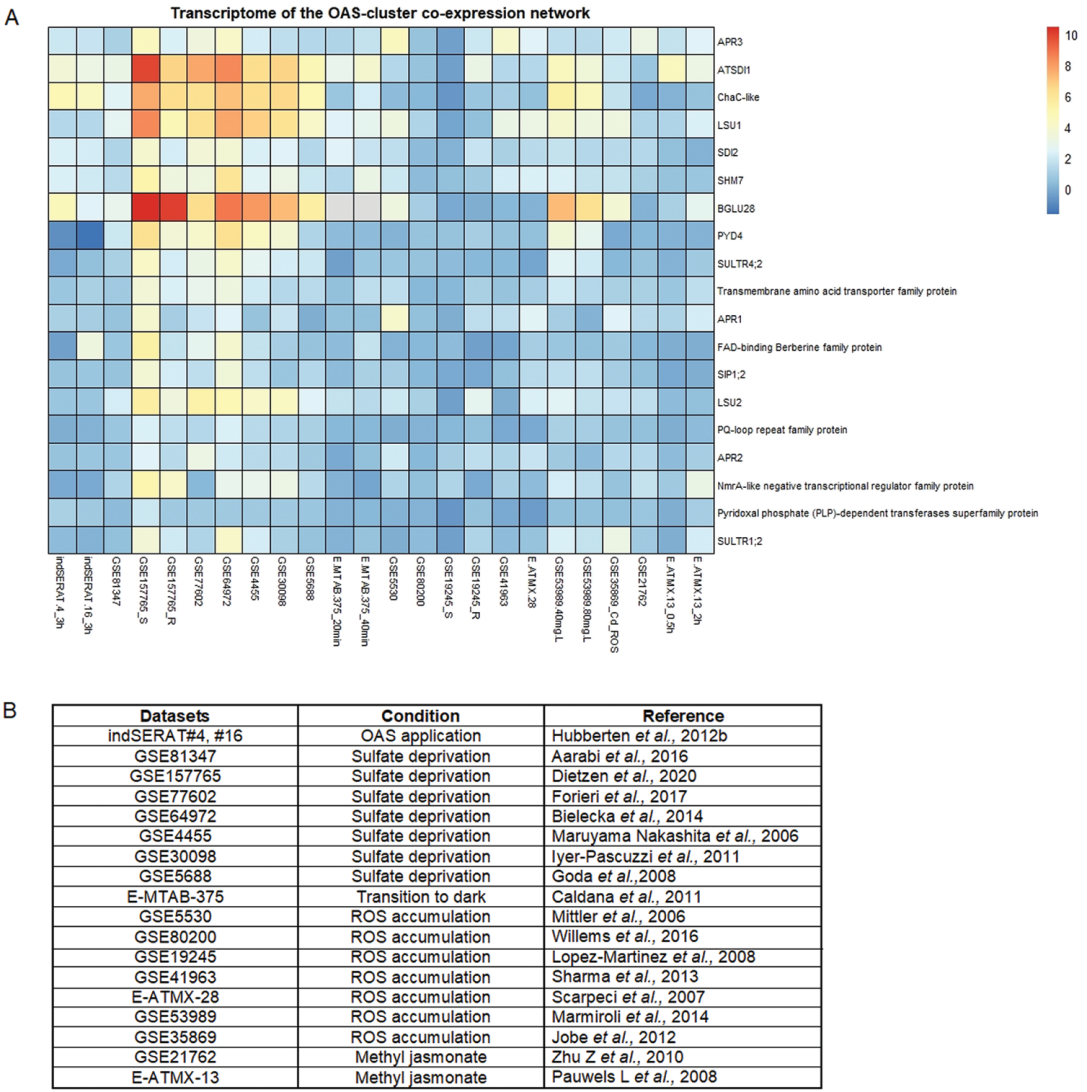
Meta-analysis depicting gene expression values of the extended OAS cluster co-expression gene network under conditions that result in OAS accumulation. (A) The data were collected from the available transcriptomic datasets (microarray and RNA-seq), using the Gene Expression Omnibus (GEO DataSets) provided by the NCBI. All the values are relative to the control—full nutrient conditions of each experiment—and log2 expression values. The heatmap was designed in R, using the Pheatmap function. No clustering was chosen. A few genes, such as those encoding hypothetical protein, *SERAT3;2*, and *LSU3*, are not included in the meta-analysis presented, since these genes were not found to have an available representative public ID in the GPL198 platform. (B) Available transcriptomic dataset IDs, which were used to design the heatmap in (A). For each dataset, not only the condition of treatment or growth is mentioned, but also the respective reference.

Under sulfur deprivation, where OAS is strongly induced, the transcripts of OAS-responsive genes such as *SDI1* and *SDI2* are drastically increased (Hirai *et al.*, 2003; Howarth *et al.*, 2009; Hubberten *et al.*, 2012b; Dong *et al.*, 2017), (Fig. 1). SDIs are also induced in SERAT-overexpressing Arabidopsis plants accumulating OAS despite sulfate-sufficient growth conditions, showing that OAS alone is able to induce the OAS cluster genes, among them SDIs (Hubberten *et al.*, 2012b) (Fig. 1). SDI proteins contain a tetratricopeptide repeat (TPR) domain, which is known to mediate protein–protein interactions (Aarabi *et al.*, 2016). It has been shown that SDI1 and SDI2 act as major repressors of GSL biosynthesis in sulfur deprivation conditions (Aarabi *et al.*, 2016). SDI1 is localized in the nucleus and forms a complex negatively affecting the transcription factor (TF) MYB28 (Aarabi *et al.*, 2016) which promotes aliphatic GSL biosynthesis (Hirai *et al.*, 2003; Gigolashvili *et al.*, 2007b). Additionally, it was shown through transient transactivation assays that SDI1 inhibits the MYB28-mediated transactivation of the promoters of two aliphatic GSL biosynthetic genes, *CYP79F1* and *CYP83A1* (Aarabi *et al.*, 2016). SDI2 lacks a nuclear localization signal, but it complements an *sdi1* knockout line, indicating that it is able to move into the nucleus. Presumably its protein–protein interaction capacity might recruit a carrier protein assisting SDI2’s access to the nucleus. From RNA sequencing data available under accession number GSE157765 (Dietzen *et al.*, 2020), it can be deduced that the basal expression of *SDI1* under complete nutrient conditions is significantly lower than that of *SDI2* (Fig. 1). This possibly indicates that *SDI2* provides basic cellular functions while *SDI1* is responsible for the strong response to stress, such as sulfur deficiency and OAS accumulation. Further research on *SDI2* needs to be performed in order to determine its basal functions under non-stress conditions. Recent findings for SDI1 demonstrated that besides inhibition of GSL biosynthesis, it down-regulates, developmentally or in response to sulfate deprivation, the biosynthesis of sulfur-rich 2S seed storage proteins in Arabidopsis seeds. SDI1 forms a protein complex with MYB28 and MYC2, which binds, for example, to the *At2S4* gene promoter (Aarabi *et al.*, 2021). It can be speculated that GSL inhibition is an acquired feature in *Brassicaceae*, while its control of seed protein composition is a feature of all seed plants.

### LSU-like family—LSU1

The LSU family in Arabidopsis consists of four members: *LSU1* (AT3G49580), *LSU2* (AT5G24660), *LSU3* (AT3G49570), and *LSU4* (AT5G24655). With the exception of LSU4, the other LSUs are induced under sulfur deprivation conditions (Hubberten *et al.*, 2012a; Sirko *et al.*, 2015; Maruyama-Nakashita, 2017; Li *et al.*, 2020) (Fig. 1). LSU-like proteins are involved in plant responses to nutrient changes, such as sulfur deprivation (Dietzen *et al.*, 2020), salt stress, or plant immune responses (Sirko *et al.*, 2015), with their transcripts accumulating under these conditions (Garcia-Molina *et al.*, 2017). Loss-of-function mutants of LSU proteins showed sensitivity towards nutrient deficiency, salinity, or heavy metal toxicity, indicating a widespread involvement of LSU family members in plant stress responses (Garcia-Molina *et al.*, 2017). LSU proteins are involved in regulating cellular degradation processes and might interact with E3 ubiquitin ligases, chaperons, and the NBR1 receptor which are involved in autophagy (Sirko *et al.*, 2015). Due to their involvement in diverse stress response pathways, further research is needed to fill the knowledge gap around LSUs.

### SHM7/MSA1

*SHM7* is a serine hydroxymethyltransferase 7 gene, also recently identified in a mutant screen study as ‘more sulfur accumulation 1’, *MSA1* (AT1G36370). *MSA1/SHM7* is among the genes that have regulatory functions under sulfur deprivation conditions and are strongly induced by OAS accumulation (Hubberten *et al.*, 2012b) (Fig. 1). Huang *et al.* (2016) used β-glucuronidase (GUS) and green fluorescent protein (GFP) constructs for MSA1 to identify the tissue expression of MSA1 and its subcellular localization. *MSA1* is highly expressed under sulfur deprivation in roots and leaves relative to full nutrient control conditions (Dietzen *et al.*, 2020) (Fig. 1). MSA1 is localized in the nucleus and its localization is unaffected by the sulfur status (Huang *et al.*, 2016). Loss-of-function mutants of *MSA1* show a reduction of *S*-adenosylmethionine (SAM) levels, by inhibition of folate biosynthesis, which as a result reduces the DNA methylation levels, leading to a sulfur deprivation response (Huang *et al.*, 2016). In *msa1* under full nutrient conditions, genes such as *SULTR1;1*, *SULTR1;2*, *APR3*, and *ATPS4* displayed increased expression levels, suggesting that the promoters of these genes were unmethylated, thus mimicking the state under sulfur-deprived growth conditions (Huang *et al.*, 2016). It is further speculated that histone methylation and histone acetylation might play an important role in sulfur homeostasis (Huang *et al.*, 2019) which would provide a gross regulation of various pathways. SHM7, unlike other members of the SHM family, does not display tetrahydrofolate biosynthetic activity. SHM7 has further been implicated to function, for example, during fruit ripening and gametogenesis, and to be localized in the nucleus (reviewed in Nogués *et al.*, 2022). In summary, *SHM7* is likely to be involved in epigenetic modifications in response to sulfate stress and OAS accumulation, probably affecting the regulation of primary sulfate metabolism, but it is also probably functional in further developmental processes.

### GGCT2;1 or ChaC-like family

*GGCT2;1* (AT5G26220) or *ChaC-like* is another member of the OAS cluster genes (Hubberten *et al.*, 2012b), which is sharply increased upon sulfur deprivation and when OAS accumulates (Fig. 1). *GGCT2;1* then displays high expression in roots compared with shoots (Joshi *et al.*, 2019). A further study using long-term growth on reduced sulfate-containing medium revealed strong induction in roots as well in shoots (Dietzen *et al.*, 2020). In inducible SERAT Arabidopsis plants, *GGCT2;1* is significantly induced, where OAS internally accumulates (Hubberten *et al.*, 2012b) (Fig. 1). GGCT2;1 is localized in the apoplast (Ferretti *et al.*, 2009) where it initiates glutathione (GSH) degradation to l-glutamate, l-cysteine, and l-glycine in the γ-glutamyl cycle (Joshi *et al.*, 2019; Ito *et al.*, 2022). In root tips of the *ggct2;1* mutant, the GSH content was increased compared with Col-0, corroborating that GGCT2;1 is involved in GSH degradation (Joshi *et al.*, 2019). GGCT2;1 affects root architecture, in correlation with GSH degradation, as *ggct2;1* under sulfur deprivation conditions demonstrates increased primary root length and less suppression of lateral root growth than Col-0 plants (Joshi *et al.*, 2019). GGCT2;1 participates through GSH degradation in the cellular responses during abiotic stress, such as toxic metal detoxification (Paulose *et al.*, 2013) or ROS accumulation (Dorion *et al.*, 2021). *GGCT2;1* transcripts accumulate under salinity stress (Gong *et al.*, 2005). Recently it was shown that the cytosolic γ-glutamyl peptidases (GGP1 and GGP3) exhibit GSH-degrading activity similar to GGCT2;1. Under full nutrient conditions, the GSH concentration in *ggp1-1* was significantly higher relative to Col-0 and *ggct2;1*, and, interestingly, this mutant accumulated OAS. This indicated that under full nutrient conditions GGP1 and probably also GGP3 degrade GSH, while surprisingly the more energy-consuming pathway via *GGCT2;1* is induced under sulfate deprivation (Ito *et al.*, 2022). It can be speculated that the resulting 5-oxoproline from the GGCT2;1 branch might be used to contribute to biotic stress responses (Fonseca *et al.*, 2021), alleviating the reduction of GSL accompanying sulfur depletion. At the same time sulfur from GSH is recycled to primary metabolism while in parallel SDIs reduce *de novo* biosynthesis of GSL.

### APR family—APR3

The APR family (*APR1*: AT4G04610, *APR2*: AT1G62180, and *APR3*: AT4G21990) are considered to be key enzymes not only for sulfate assimilation in higher plants but also for the nitrate assimilation pathway and diurnal rhythm (Kopriva *et al.*, 1999; Lee *et al.*, 2011). All three APR isoforms demonstrate decreased enzyme activity under darkness (Kopriva *et al.*, 1999). APR3 is found to be exclusively localized in chloroplasts where it catalyzes the reduction of APS to sulfite by transferring two electrons (Koprivova *et al.*, 2008). APR activity is increased by OAS (Lee *et al.*, 2011). Further, the TF HY5 (AT5G11260) which coordinates nitrogen and sulfur assimilation, is a regulator of APR expression (Lee *et al.*, 2011). Additionally, APR activity and mRNA levels of all three APR isoforms increased under treatment with NaCl. APR transcripts were unaffected in mutants deficient in abscisic acid (ABA) synthesis while treatment of plants with ABA did not alter the mRNA levels of APR, showing that APR is regulated by salt stress in an ABA-independent manner (Koprivova *et al.*, 2008). In summary, APR is integrating various metabolic and stress inputs to coordinate sulfate assimilation, by potentially increasing flux through the assimilation and reduction pathway. APRs are induced under sulfur deprivation (Dietzen *et al.*, 2020), but not significantly up-regulated by OAS application (Fig. 1) (Hirai *et al.*, 2003; Hubberten *et al.*, 2012b).

## Additional genes potentially playing a role in the OAS response

**Fig. 2.**
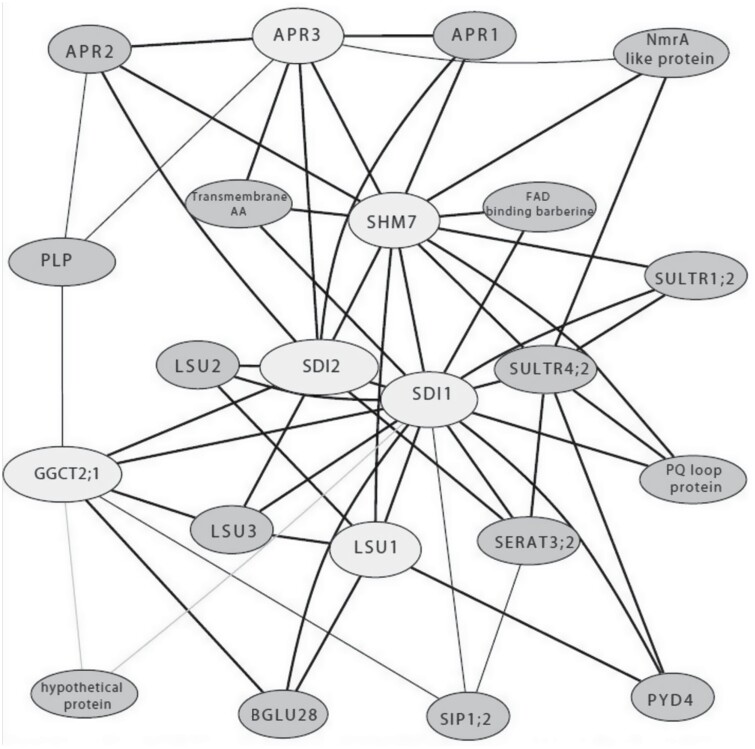
The extended OAS cluster co-expression gene network from ATTED-II. The six OAS cluster genes (light gray color) were used as query genes on the ATTED-II database. The ATTED-II contains data derived from RNA-seq (Ath-r.c5-0 platform) and microarray (Ath-m.c9-0). An additional 16 genes (dark gray color) were found to be stably co-regulated with the six core OAS cluster genes. Among them, several genes are known to be associated with sulfur metabolism and response to sulfur deprivation, such as APRs, LSUs, SULTRs, and SERAT3;2. The *z*-score is a factor which indicates the stability of the co-expression. The thicker line, which connects two genes, indicates a higher *z*-score displaying a higher degree of co-expression between the two genes. The thinner the line is, the lower the *z*-score is. A lower *z*-score indicates lower stability of co-expression of the two genes.

ATTED-II is a gene co-expression database for plant species, Arabidopsis included, based on publicly available RNA-seq-derived data from the Ath-r.c5-0 platform (14 741 runs) and microarray data from Ath-m.c9-0 (12 686 chips) (Obayashi *et al.*, 2022). Using ATTED-II, a co-expression network was built using as a query the above-mentioned ‘OAS cluster genes’ (Hubberten *et al.*, 2012b) which comprise 22 genes (Fig. 2) being stably co-expressed under many diverse conditions. Genes are recruited to the network when co-expressed with at least two genes from the query gene list. As displayed in Table 1, the core OAS cluster genes display the highest connectivity within the network. This analysis obviously does not only identify OAS-responsive genes, which in turn might allow the discernment of co-regulation properties.

**Table 1.** The new extended OAS cluster co-expression gene network from ATTED-II

Gene name	AGI code	No. of genes connected	GO terms	Reference
FAD binding barberine	AT4G20820	2	Sulfur metabolism	Depuydt and Vandepoele (2021)
SDI2	AT1G04770	8	Sulfur metabolism	Maruyama-Nakashita *et al.* (2005)
SULTR4;2	AT3G12520	7	Sulfur metabolism	Kataoka *et al.* (2004)
APR3	AT4G21990	7	Sulfur metabolism	Bick and Leustek. (1998)
LSU1	AT3G49580	6	Sulfur metabolism	Maruyama-Nakashita *et al.* (2005); Sirko *et al.* (2015)
LSU3	AT3G49570	4	Sulfur metabolism	Depuydt and Vandepoele (2021)
APR2	AT1G62180	4	Sulfur metabolism	Setya *et al.* (1996)
LSU2	AT5G24660	3	Sulfur metabolism	Sirko *et al.* (2015)
APR1	AT4G04610	3	Sulfur metabolism	Setya *et al.* (1996); Bick *et al.* (1998); Koprivova *et al.* (2000)
SULTR1;2	AT1G78000	3	Sulfur metabolism	Rouached *et al.* (2005, 2008)
ChaC-like/GGCT2;1	AT5G26220	7	Sulfur metabolism, glutathione process	Paulose *et al.* (2013)
SERAT3;2	AT4G35640	4	Sulfur metabolism, cysteine	Kawashima *et al.* (2005)
SDI1	AT5G48850	16	Sulfur metabolism, carbon metabolism, transcription	Maruyama-Nakashita *et al.* (2005)
SHM7	AT1G36370	12	Sulfur metabolism, carbon metabolism, methionine process	Huang *et al.* (2016)
NmrA-like protein	AT1G75280	3	Response to oxidative stress biosynthesis	Babiychuk *et al.* (1995)
PQ loop repeat family protein	AT5G40670	3	cysteine biosynthesis	Gaudet *et al.* (2011)
BGLU28	AT2G44460	3	Carbon metabolism, glucosinolate hydrolysis	Gaudet *et al.* (2011)
Pyridoxal phosphate (PLP) protein	AT1G77670	3	Carbon metabolism, transaminase activity	Depuydt and Vandepoele (2021)
PYD4	AT3G08860	3	Response to nitrogen, transaminase activity	Zrenner *et al.* (2009)
SIP1;2	AT5G18290	3	Transporter activity	Ishikawa *et al.* (2005)
Transmembrane amino acid protein	AT3G56200	3	Amino acid transporter activity	Gaudet *et al.* (2011)
hypothetical protein	AT2G32487	2	Response to ABA	Depuydt and Vandepoele (2021)

Summarizing table demonstrating information about the extended OAS cluster co-expression gene network from ATTED-II. The table provides information about the number of the genes to which each gene is connected in the network depicted in Fig. 2. *SDI1* shows the highest connectivity between the network, connected with 16 genes out 22. This means that *SDI1* is co-expressed with most of the genes existing in the network, throughout many transcriptomic experiments in *A. thaliana*. Additionally, information is provided about the GO terms of each gene. Most of the genes are involved in sulfur metabolism and sulfur response (*SULTR*, *SERAT3;2*, *LSU*, *SDI*, *APR*). Additionally, a few seem to be involved in carbon metabolism or GSL regulation (*BGLU28*), cysteine biosynthesis (*PQ loop repeat family protein*), oxidative stress (*NmrA-like protein*), or ABA response (*hypothetical protein*).

### The OAS cluster co-expression network

A majority of the genes (14) that are present in the OAS cluster co-expression network are known to be involved in sulfur metabolism. Gene Ontology (GO) enrichment analysis (Table 1) using ATTED (Obayashi *et al.*, 2022) and TAIR (Berardini *et al.*, 2015) revealed biological processes except for sulfur metabolism. These include carbon and nitrogen metabolism (metabolic processes) or terms involved in stress response, such as oxidative stress or ABA (Table 1). It is known that sulfate availability affects the ABA content in plant tissues, as the ABA level is reduced in sulfate-deprived plants (Cao *et al.*, 2014). This explains why genes involved in stresses or responsive to different ABA levels, such as the *hypothetical protein* (AT2G32487), are included in the OAS cluster ATTED network. The sulfate assimilation pathway and the carbon and nitrate pathways converge at the level of cysteine synthesis. In the plant cell, the pathways of carbon assimilation, through the Calvin cycle, of nitrate assimilation, and of sulfate assimilation co-influence one another (Jobe *et al.*, 2019). The inter-relationship between those pathways is very strong since sulfate deprivation reduces nitrate uptake and carbon assimilation, and vice versa. The sensors and mechanisms of the connection of these three pathways are poorly understood (Koprivova and Kopriva, 2014). It is likely that the pathway genes could transcriptionally respond in concert if any changes occur in one of the three pathways. Hence, the GO enrichment analysis of the OAS cluster co-expression network (Table 1) displays genes involved in nitrogen response (*PYD4*) or carbon metabolism (*PLP*, *BGLU28*, and *SDI1*).

#### BGLU28

A similar response as the OAS cluster genes under sulfur deprivation or OAS accumulation is displayed by β-glucosidase 28 (*BGLU28*) (AT2G44460), which is considered to be a sulfur deprivation marker (Zhang *et al*., 2014). BGLU28 is involved in GSL catabolism under sulfur deprivation conditions by hydrolyzing GSL resulting in d-glucose and sulfate in order to recycle sulfate from GSL for primary metabolism (Nikiforova *et al.*, 2004; Maruyama-Nakashita *et al.*, 2005, 2006). Zhang *et al*. (2020) proved this hypothesis by using a double knockout mutant of *BGLU28* and *BGLU30* (AT3G60140), encoding another main β-glucosidase and likewise induced by sulfate deprivation. The double knockout *bglu28/30* displayed growth retardation and reduced metabolic performance under sulfur deprivation relative to Col-0, with GSL contents being increased and other sulfur-containing compounds such as GSH, cysteine, or sulfate being reduced (Zhang *et al*., 2020). In support of the GSL recycling function, it could be shown that labeled ^34^S from GSL was allocated to primary sulfur metabolism as substrate, eventually ending up in, for example, cysteine or GSH (Sugiyama *et al.*, 2021). These results prove not only the role of BGLU28 in GSL catabolism but also the role of GSL as a sulfur reservoir (Sugiyama *et al.*, 2021). *BGLU28* displays high connectivity within the OAS cluster co-expression network (Fig. 2) and it is strongly induced under sulfur deprivation conditions (Dietzen *et al.*, 2020) (Fig. 1) but less induced in inducible *SERAT* plants or upon OAS treatment (Hirai *et al.*, 2003; Hubberten *et al.*, 2012b) (Fig. 1). *BGLU30* is not OAS induced, though it is also a sulfate deprivation-inducible gene, and both *BGLU28* and *BGLU30* are controlled by SLIM1 (Dietzen *et al.*, 2020). This again indicates that although OAS accumulates under sulfur deprivation conditions, there must be additional signals for sulfate deprivation-specific gene regulation.

#### SERAT family

*SERAT3;2* is part of the co-expression network (Fig. 2; Table 1). Of the five *SERAT* genes, *SERAT1;1* (At5g56760), *SERAT2;1* (At1g55920), *SERAT2;2* (At3g13110), *SERAT3;1* (At2g17640), and *SERAT3;2* (At4g35640), each has a different transcriptional response to certain conditions (Watanabe and Hoefgen, 2017). It was shown that *SERAT* group III genes are highly induced under sulfur deprivation, while group II is induced under oxidative stress and prolonged sulfate deprivation (Watanabe *et al.*, 2015; Watanabe and Hoefgen, 2017; Dietzen *et al.*, 2020); group I does not respond to tested conditions. The variability in the transcript responses of the *SERAT* genes under sulfur deprivation might indicate that the plant organism balances the OAS production and responds to the need for OAS transport to different cellular compartments (Watanabe and Hoefgen, 2017).

#### Sulfate transporters (SULTRs)

*SULTR4;2* and *SULTR1;2* are part of the OAS cluster co-expression network (Fig. 2; Table 1). Plants take up sulfate through their root system with the help of root high-affinity sulfate transporters SULTR1;1 (AT4G08620) and SULTR1;2 (AT1G78000) (Takahashi *et al.*, 2000, 2011; Yoshimoto *et al.*, 2007). SULTR1;1 and SULTR1;2 are the main transporters involved in sulfate assimilation and are increased under sulfur-deprived conditions (Fig. 1) at transcriptional and protein levels (Takahashi *et al.*, 2000; Yoshimoto *et al.*, 2007). SULTR2;1 (AT5G10180) and SULTR2;2 (AT1G77990) are suggested to transfer sulfate from the roots to the shoots, and SULTR2;1 also controls the sulfate transfer into the developing seeds (Takahashi *et al.*, 2011). Group III sulfate transporters have been shown to be expressed mainly in leaves (Takahashi *et al.*, 2000). SULTR3;1 (AT3G51895) is responsible for the sulfate uptake into the chloroplasts (Cao *et al.*, 2014). SULTR4;1 (AT5G13550) and SULTR4;2 (AT5G13550) are tonoplast-localized transporters and coordinate the release of sulfate from the vacuoles (Takahashi *et al.*, 2011). *SULTR4;1* and *SULTR4;2* are highly induced under sulfur deprivation (Takahashi *et al.*, 2000, 2011; Cao *et al.*, 2014) (Fig. 1). It is known that SULTRs, group I and IV, respond to OAS accumulation in inducible SERAT plants and under OAS application (Hirai *et al.*, 2003; Hubberten *et al.*, 2012b).

#### PYD4

*PYD4* (AT3G08860) belongs to the aminotransferase gene family, and it functions as an alanine:glyoxylate aminotransferase/β-alanine:pyruvate aminotransferase (Parthasarathy *et al.*, 2019). *PYD4* is up-regulated in response to osmotic stress and has been shown to be putatively involved in β-alanine metabolism (Parthasarathy *et al.*, 2019). Studies have identified that PYD4 is involved in multiple processes in plants, such as changes in light and carbon availability (Parthasarathy *et al.*, 2019). PYD4 is localized in the mitochondria or the peroxisome (Niessen *et al.*, 2012) and it is down-regulated in the microarray of Hubberten *et al.* (2012b), where OAS is induced internally; however, in sulfur deficiency microarrays and RNA-seq, *PYD4* is co-regulated with the OAS cluster genes (Fig. 1). It is connected with *SDI1*, *LSU1*, and *SULTR4;2* in the OAS cluster co-expression network (Fig. 2), and controlled by SLIM1 (Table 2). *PYD4*, like *BGLU30*, seems to be sulfate deprivation responsive rather than OAS responsive.

**Table 2.** SLIM1 binding in the promoters and regulation of the new extended OAS cluster co-expression gene network from ATTED-II

Gene name	AGI code	Dap-seq EIL3	MN 2006 EIL3	EIL3-Dietzen and -S
ATSDI1	AT5G48850	Yes	Yes	Yes
SHM7	AT1G36370	Yes	Yes	Yes
SDI2	AT1G04770	Yes	Yes	Yes
SULTR4;2	AT3G12520	Yes	Yes	Yes
ChaC-like/GGCT2;1	AT5G26220	Yes	Yes	Yes
APR3	AT4G21990	Yes	No	Yes
LSU1	AT3G49580	Yes	Yes	Yes
LSU3	AT3G49570	Yes	No	Yes
APR2	AT1G62180	Yes	No	Yes
SERAT3;2	AT4G35640	No	–	Yes
LSU2	AT5G24660	Yes	No	Yes
APR1	AT4G04610	Yes	No	Yes
SULTR1;2	AT1G78000	Yes	Yes	Yes
PYD4	AT3G08860	Yes	Yes	Yes
Transmembrane AA	AT3G56200	Yes	No	Yes
BGLU28	AT2G44460	Yes	Yes	Yes
SIP1;2	AT5G18290	No	–	Yes
PQ loop repeat family	AT5G40670	No	Yes	Yes
FAD binding barberine	AT4G20820	Yes	No	Yes
Pyridoxal phosphate (PLP)	AT1G77670	Yes	No	Yes
NmrA-like negative transcriptional regulator	AT1G75280	No	Yes	Yes
hypothetical protein	AT2G32487	Yes	No	Yes

Information about the regulation of the extended OAS cluster co-expression gene network from ATTED-II by EIL3/SLIM1. We used three different experimental approaches to demonstrate the binding and regulation of SLIM1 at the promoters of the extended OAS cluster co-expression gene network from ATTED-II genes. First, we used the data from Dap-seq by O’Malley *et al.* (2016) which showed direct binding of SLIM1 at the promoters of all the genes except *SERAT3;2*, *SIP1;2*, *PQ loop family protein*, and *NmrA like negative transcriptional regulator*. Microarray analysis by Maruyama-Nakashita *et al.* (2006) indicates that SLIM1 regulates the majority of the genes, with few exceptions. Interestingly, the genes to which SLIM1 was found not to bind (Dap-seq), are not the same as those that seem not to be regulated by SLIM1 (MN). Moreover, RNA-seq by Dietzen *et al.* (2020) indicates that SLIM1 can regulate all the genes under sulfur deprivation conditions.

#### SIP1;2

*SIP1;2* (AT5G18290) encodes an aquaporin and expressed in all Arabidopsis tissues except dry seeds. It is localized in the endoplasmic reticulum (ER) membrane and has a water channel activity (Ishikawa *et al.*, 2005). SIP1;2 is involved in controlling the volume and morphology of the ER lumen and the concentration of ions in the ER (Ishikawa *et al.*, 2005). *SIP1;2* is slightly induced in OAS-accumulating plants (Hubberten *et al.*, 2012b) and under sulfur deficiency (Dietzen *et al.*, 2020) (Fig. 1).

### Additional members of the co-expression network from ATTED

The OAS cluster co-expression network contains four genes that have not been studied extensively before. These are *LSU3* (AT3G49570), *FAD binding berberine family protein* or *AtBBE18* (AT4G20820), *pyridoxal phosphate* (PLP)-dependent transferase (AT1G77670), and the *hypothetical protein* (AT2G32487) (Fig. 2; Table 1). Depuydt and Vandepoele (2021) identified the functional relationship of these three genes to sulfur metabolism and/or OAS. *LSU3* was found to respond to sulfur deprivation and other stresses such as salt stress, ABA, wounding, or exposure to fungi. This agrees with the previous predictions and results for the involvement of the LSU family in stresses (Sirko *et al.*, 2015; Garcia-Molina *et al.*, 2017). The *FAD binding berberin* was annotated with only one GO term, sulfur metabolic process, without any indication on its molecular or physiological function (Table 1) (Depuydt and Vandepoele, 2021). *At*BBE18 has been characterized as a biomass regulator and was shown to be important for salt stress tolerance (Daniel *et al.*, 2016). The pyridoxal phosphate (PLP)-dependent transferase protein is associated with cellular responses to stress, carboxylic acid metabolic processes, and seed development, while the hypothetical protein AT2G32487 is involved in the ABA response (Depuydt and Vandepoele, 2021). Reduced sulfate availability reduces ABA contents in plant tissues (Cao *et al.*, 2014) while ABA accumulates under abiotic stresses such as salt or drought. High concentrations of NaCl result in sulfate content reduction (Hongqiao *et al.*, 2021). The inclusion of *LSU3*, *FAD binding berberine*, and *(PLP)-dependent transferase* in the OAS cluster co-expression network indicates a relationship between the pathways of sulfate, and ABA or salt.

## Functional relationships between the genes of the OAS cluster co-expression network

It is striking that the genes of the expanded OAS cluster co-expression network are involved in various metabolic processes with the major one being sulfur metabolism (Table 1). These processes are directly connected with the sulfur pathway (cysteine, methionine, GSH, and GSL) or seemingly unrelated (ABA or ROS response, and carbon, nitrogen, and amino acid metabolism). Their co-expression, however, suggests that there might be physiological and functional relationships between the genes involved in these pathways and a need for coordinated regulations. When exposed to sulfur deficiency, plants alter morphological and physiological processes, in which the above-mentioned genes are involved. The initial step of the sulfur assimilation pathway (sulfur sufficiency or deprivation) is the absorption of any sulfate molecule from the soil by the SULTR1 transporters (Li *et al.*, 2020). After a series of enzymatic reactions (see Box 2), sulfate is incorporated into cysteine, and some of the genes shown in Fig. 2, are involved in these steps: SULTR4;2 translocates sulfur from the vacuoles where its stored in order to cover the demands of sulfate-deficient plants (Kataoka *et al.*, 2004). In parallel, APR3 drives sulfate reduction for primary metabolism rather than sulfate processes catalyzed by SERATs (SERAT group III) (De Kok *et al.*, 2017; Watanabe and Hoefgen, 2017). Sulfur deprivation activates recycling processes of sulfate-containing molecules such as as GSH or GSLs by GGCT2;1 or BGL28 (Paulose *et al.*, 2013); Zhang *et al*., 2020; Ito *et al.*, 2022) or reduces their biosynthesis through inhibiting the MYB28 regulatory activity by SDI genes (Aarabi *et al.*, 2016, 2020). Further, general degradatory processes such as autophagy through *LSU* genes (*LSU1* and *LSU3*) are induced (Sirko *et al.*, 2015; Dong *et al.*, 2022). Methylation by MSA1 very generally affects diverse processes in response to sulfate deprivation (Huang *et al.*, 2016, 2019). Lastly, ROS and OAS accumulation often appear linked (Dorion *et al.*, 2021) and it might be speculated that OAS serves as a signal under sulfate-deprived conditions but also under sulfate-sufficient conditions when ROS levels increase together with OAS (Hubberten *et al.*, 2012b; Aarabi *et al.*, 2020). As such, the co-expressed genes are presumably part of a complex regulatory network integrating diverse inputs, eventually regulating plant cell homeostasis. This is reflected by the fact that the promoters of OAS cluster genes analyzed so far contain diverse sets of *cis*-elements, and various TFs have been identified to affect their respective expression (Rakpenthai *et al.*, 2022).

## OAS, sulfur metabolism, and their connection with hormones

**Fig. 3.**
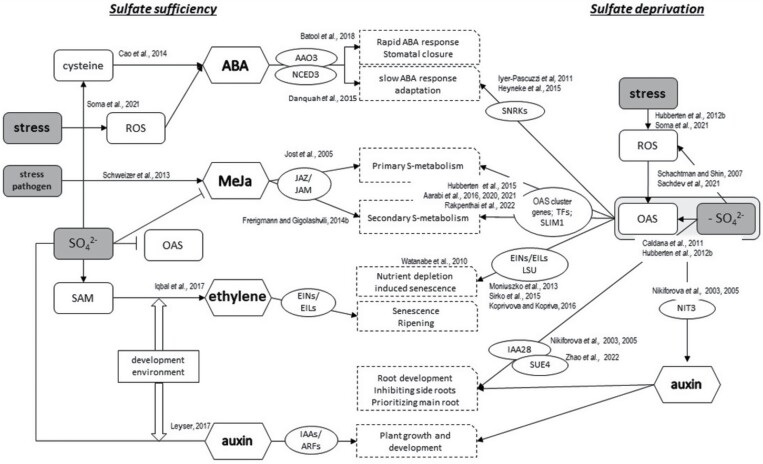
Scheme of interaction and crosstalk of sulfate metabolism and various signal hormones, such as ABA, MeJa, ethylene, and auxin. Arrows with arrowheads represent induction while arrows with blunt ends represent negative regulation. The outcome of the hormone effect is depicted in broken border boxes. References are reported in the figure with respective to each pathway.

Plant hormones are regulators of a diverse set of physiological responses in plants. Information on the interplay of sulfate metabolism, especially under conditions of sulfate deprivation, is still fragmented. Systematic studies on the dynamics and tissue specificity of hormone responses are still lacking. However, available research allows us to offer a first overview on the topic (Fig. 3). However, at this level, it is not yet possible to differentiate between the effects of sulfate deprivation and its potential direct effects, and the signals involved and those exerted by OAS. ABA is one of the key regulators of stress responses (Soma *et al.*, 2021). Sulfur availability and especially increased cysteine levels positively affect ABA biosynthesis (Cao *et al.*, 2014) and control stomatal closure through ABA (Batool *et al.*, 2018). In addition to this rapid response, for example to drought conditions, stress-induced ABA also fosters adaptation processes widely affecting plant physiology (Danquah *et al.*, 2015). Sulfate deprivation leads in roots to the induction of many regulatory genes, as described above. In particular, *SNRK* genes have been described to be responsive to various nutrient stresses (Iyer-Pascuzzi *et al.*, 2011; Heyneke *et al.*, 2015), among them *SNRK2* as part of the ABA core signaling module and *SNRK3.15* and *SnRK3.22* as central hubs controlling ABA-responsive genes (Lumba *et al.*, 2014). *SNRK 3.15* is induced under sulfate deprivation (Iyer-Pascuzzi *et al.*, 2011; Heyneke *et al*., 2015), when ABA does not accumulate (Cao *et al.*, 2014), possibly allowing recruitment of ABA-dependent responses under sulfate deprivation, such as nutrient depletion-induced senescence (NUDIS) (Watanabe *et al.*, 2010). OAS also accumulates upon ROS accumulation when plants are exposed to stresses (Hubberten *et al.*, 2012b) and among those stresses also upon sulfate deprivation (Schachtman and Shin, 2007; Sachdev *et al.*, 2021). LSU1 has been found to reduce ROS production under sulfur deprivation and promote stomatal closure (Garcia-Molina *et al.*, 2017), though the mechanism is as yet unclear.

Jasmonate application induces the expression of numerous genes involved in sulfur assimilation, methionine biosynthesis, SAM biosynthesis, and further sulfur-related processes (Jost *et al.*, 2005) and *SDI1* (Rakpenthai *et al.*, 2022). Transcriptome analyses of plants under sulfur-deprived conditions revealed that genes of MeJa biosynthesis are induced within 24–48 h (Hirai *et al.*, 2003; Nikiforova *et al.*, 2003; Jost *et al.*, 2005). Whether the jasmonate signaling pathway is activated by OAS or by signals such as ROS (Koo, 2018) remains to be validated. In the JAZ/JAM system (Fig. 3), the JASMONATE ZIM-domain (JAZ) and CORONATINE INSENSITIVE1 (COI1) protein complex inhibits jasmonate target gene expression. MeJa leads to polyubiquitination of JAZ, priming it for degradation and allowing other TFs such as MYC2 to access the promoter which, for example, leads to activation of GSL biosynthesis. MYC2 and MYB28 are TFs critical for inducing GSL synthesis (Schweizer *et al.*, 2013; Frerigmann and Gigolashvili, 2014). SDI1, on the other hand, inhibits GSL biosythesis through interaction with MYC2 and MYB28 (Aarabi *et al.*, 2016) which appears contradictory. *SDI* genes and OAS cluster genes act to prevent flux into secondary metabolites or to retrieve sulfur by degradation and induce primary sulfate metabolism (Aarabi *et al.*, 2020), thus reducing the plant’s capacity to react against biotrophic pathogens. MeJa induces biosynthesis of secondary metabolite such as GSL which might possibly be a mechanism to redirect sulfur to secondary metabolite biosynthesis when exposed to MeJa-inducing pathogens (Schweizer *et al.*, 2013). The exact interplay of this cross-regulatory effect needs to be further investigated. Another link between jasmonate signaling and OAS might be the basic helix–loop–helix (bHLH) TF At1g10585 that is induced by OAS and not sulfate deprivation, is a target gene of the JAZ/JAM system (Hubberten *et al.*, 2015), and is ROS responsive (Inze *et al.*, 2012).

Ethylene is a key regulator of leaf senescence and fruit ripening (Iqbal *et al.*, 2017) as well as nutrient depletion-induced senescence (Watanabe *et al.*, 2010). Ethylene is tightly linked to methionine metabolism (Moniuszko *et al.*, 2013; Sirko *et al.*, 2015; Koprivova and Kopriva, 2014) as SAM is the precursor of ethylene synthesis (Fig. 3). Members of the EIL (ETHYLENE-INSENSITIVE) TF family, such as EIN3, EIL1, EIL2 (Wawrzyńska *et al.*, 2010), or EIL3/SLIM1 have been shown to regulate sulfur-responsive genes under sulfur deprivation conditions (Maruyama-Nakashita *et al.*, 2006; Wawrzyńska and Sirko, 2016; Dietzen *et al.*, 2020), demonstrating their involvement in ethylene-responsive and sulfur-responsive gene regulation. Under sulfur deprivation conditions, ethylene accumulates in Col-0 plants. Tobacco *LSU* and *UP9C* mutants (Sirko *et al.*, 2015) accumulated significantly less ethylene than Col-0. Additionally, in *up9c*, under sulfur deprivation conditions, the transcripts of ethylene-responsive genes were significantly less expressed relative to Col-0. Hence, members of the LSU family are likely to be involved in the modulation of the ethylene signaling pathway which is crucial for the sulfur deficiency response (Sirko *et al.*, 2015) (Fig. 3). Yet, SAM levels, and hence the precursor of ethylene, massively decreased upon prolonged sulfate deprivation due to the reduced availability of methionine (Nikiforova *et al.*, 2005b) and despite the activity of the SAM/Met cycle (Büerstenbinder *et al.*, 2007). Our understanding of the interplay of ethylene, OAS, and sulfate is still lacking details to provide a resolved model.

Auxin is involved in numerous aspects of plant physiology, coordinating plant growth and development by affecting transcription through the AUX/IAA–ARF system as well as polarity in organs such as roots through directed transport by the PIN system (Leyser, 2018). It is thus not surprising that auxin-related genes have been identified to be responsive to sulfate deprivation (Nikiforova *et al.*, 2004; Dietzen *et al.*, 2020) among them induction of nitrilase 3 (*NIT3*) involved in auxin biosynthesis. Further, IAA28 has been identified as a hub in network analysis under sulfate deprivation (Nikiforova *et al.*, 2005a; Watanabe and Hoefgen, 2017) though its expression is only marginally increased (Dietzen *et al.*, 2020). IAA28 probably inhibits lateral root development in response to sulfate deprivation (Rogg *et al.*, 2001; Falkenberg *et al.*, 2008). Recently *SUE4* (sulfate utilization efficiency 4) was identified to be induced by sulfate starvation and to foster primary root elongation by interacting with PIN1 and targeting it for protein degradation (Zhao *et al.*, 2023) (Fig. 3). Both genes are consistent with the known plant phenotype of lateral root repression and enhanced primary root growth upon sulfate deprivation (Hubberten *et al.*, 2012a). This allows plants to search for sulfate-rich patches in the soil and expand the root system when exposed to sulfate.

While in general sulfate depletion or OAS accumulation might affect phytohormone accumulation, where, as stated above, sufficient data related to time and tissue distribution are missing, sulfate deprivation-derived signals or OAS directly affect hormone-related pathways (Fig. 3). Through this, existing hormone response pathways are utilized to facilitate sulfur deficiency/OAS-specific responses. In addition, it has to be noted that plant hormones interact and influence one another in a complex manner (Rubio *et al.*, 2009).

## How are OAS-responsive genes regulated?

The plant responses to sulfur deprivation conditions have been studied during the past decade (Davidian and Kopriva, 2010; Nakai and Maruyama-Nakashita, 2020; Ristova and Kopriva, 2022). Which signals are perceived and how TFs regulate these responses is still not finally resolved (Kopriva, 2006). Some progress has been achieved and suggestions provided (Maruyama-Nakashita *et al.*, 2006; Zhang *et al*., 2014; Bielecka *et al.*, 2015; Aarabi *et al.*, 2016; Huang *et al.*, 2016; Watanabe and Hoefgen, 2017, 2019; Forzani *et al.*, 2018; Rakpenthai *et al.*, 2022; Wawrzyńska *et al.*, 2022). In addition to these analyses, available databases allow the identification of TFs possibly involved in the OAS-driven response.

In this context, we scored TFs suggested to regulate the genes of the co-expression network (Fig. 2; Table 1). The Plant Regulomics database (Ran *et al.*, 2020) was used to identify TFs which bind to the promoters of the new network genes (Fig. 2; Table 1) in order to obtain information on whether these might be linked to the OAS signal. The dominant TF EIL3/SLIM1 binds to 18 of the 22 genes of the OAS co-expression network (Table 3).

**Table 3.** Transcription factors regulating the new extended OAS cluster co-expression gene network from ATTED-II

Transcription factor	AGI code	No. of genes regulated	Source	Regulation
EIL3	AT1G73730	18	DAP-seq	Ethylene and sulfate deprivation signaling
EIN3	AT3G20770	8	DAP-seq	Ethylene signaling
MYB67	AT3G12720	10	DAP-seq	Response to wounding
DTAF1	AT3G45810	7	ChIP-seq	NAD(P)H oxidase H_2_O_2_-forming activity,
ERF115	AT5G07310	7	ChIP-seq	Ethylene signaling
HB7	AT2G46680	7	ChIP-seq	Drought response and ABA
NFYB2	AT5G47640	7	ChIP-seq	Response to nutrient levels
E2Fa	AT2G36010	7	ChIP-seq	E2F pathway
NRPE1	AT2G40030	6	ChIP-seq	DNA methylation, defense response to fungus
BBM	AT5G17430	6	ChIP-seq	Lateral roof formation
HY5	AT5G11260	6	ChIP-seq	Anthocyanin accumulation in far-red light
NFYC2	AT1G56170	5	ChIP-seq	Response to nutrient levels
PIF4	AT2G43010	6	ChIP-seq	Shade avoidance response, response to nutrient levels
MYB3	AT1G22640	5	ChIP-seq	Phenylpropanoid biosynthesis gene expression
MYB related family	AT3G10580	5	DAP-seq	–
RVE8	AT3G09600	7	DAP-seq	Regulation of the circadian clock by modulating the pattern of histone 3 (H3) acetylation, involved in heat shock response
PIF3	AT1G09530	6	ChIP-seq	Binds to anthocyanin biosynthetic genes in a light- and HY5-independent fashion, regulation of photosynthesis, light reaction
LHY	AT1G01060	6	ChIP-seq	Circadian rhythm along with another Myb transcription factors
HB6	AT2G22430	6	ChIP-seq	Hormone responses in Arabidopsis such as ABA
CCA1	AT2G46830	4	ChIP-seq	Circadian rhythms, long-day photoperiodism, flowering
DTAF2	AT5G50360	6	ChIP-seq	ABA signaling

Transcription factors (TFs) which are suggested by Plant Regulomics to bind at the promoters of the extended OAS cluster co-expression gene network from ATTED-II. The table demonstrates the number of the genes of the extended OAS cluster co-expression gene network from ATTED-II on which a particular TF is bound on their promoters. EIL3/SLIM1 is the TF which binds to the majority of the gene promoters. Another TF binding at many promoters is MYB67. Information about the binding of the TFs was also provided by Plant Regulomics. Dap-seq (DNA affinity purification sequencing) or Chip-seq (ChIP sequencing) are the molecular experiments proving the TF binding at the promoters. With the help of the TAIR tool, the conditions in which those TFs are involved or regulated by were identified and provided.

EIL3/SLIM1 is involved in the sulfate deprivation signaling pathway and is a central transcriptional regulator of plant sulfate metabolism (Maruyama-Nakashita *et al.*, 2006; Wawrzyńska *et al.*, 2010, 2022; Kawashima *et al.*, 2011; Wawrzyńska and Sirko, 2014, 2016; Dietzen *et al.*, 2020; Rakpenthai *et al.*, 2022), by controlling many sulfate deprivation response genes. EIL3/SLIM1 binds to the UPE box, the TEBS element, and the SURE element, which are present in many sulfate- and OAS-responsive genes (Maruyama-Nakashita *et al.*, 2005; Wawrzyńska *et al.*, 2010; Rakpenthai *et al.*, 2022). In addition to binding properties as displayed in the Plant Regulomics database, transcriptional regulation of OAS cluster genes and OAS network genes could be shown using transcriptome data (Maruyama-Nakashita *et al.*, 2006; O’Malley *et al.*, 2016; Dietzen *et al.*, 2020) (Table 2). Interestingly, SLIM1 transcriptional levels do not alter under sulfur deprivation (Wawrzyńska *et al.*, 2010; Rakpenthai *et al.*, 2022) or OAS treatment (Hubberten *et al.*, 2012b), which indicates that SLIM1’s activity is affected by post-transcriptional and/or post-translational modifications. Further, it has been indicated that EIL3/SLIM1 might conditionally also act as a repressor or as an enhancer of target gene expression, for example under sulfate deprivation or arsenic treatment (Jobe *et al.*, 2019; Dietzen *et al.*, 2020), which might explain the differences in the identified transcriptional control patterns between different experiments (Table 2). Despite its early detection and prominent role the functions of EIL3/SLIM1, its interactions with other regulators, and its post-transcriptional/post-translational properties are not yet entirely resolved.

Further ethylene-responsive TFs have been suggested to regulate sulfate deprivation metabolism. EIN3 is a regulator of the ethylene pathway and interacts with EIL3/SLIM1 (Wawrzyńska and Sirko, 2016). EIN3 binds to the promoters of eight genes of the co-expression network (Table 3) and has been shown to form heterodimers with EIL3/SLIM1, preventing SLIM1 binding of the UPE-box (Wawrzyńska and Sirko, 2016). EIL1 (AT2G27050) has been shown to regulate numerous sulfate deprivation genes in concert with EIL3/SLIM1 or independently (Dietzen *et al.*, 2020). EIL1 was not identified in the Plant Regulomics dataset, leading to the conclusion that EIL1 might exert its regulatory function through protein–protein interaction with other TFs (such as EIL3/SLIM1) rather than itself binding directly to the promoter.

MYB TFs are known to be involved in numerous plant regulatory processes (Dubos *et al.*, 2010). With respect to sulfur metabolism they have been identified to be involved in GSL biosynthesis regulation (Celenza *et al.*, 2005; Gigolashvili *et al.*, 2007a; Frerigmann and Gigolashvili, 2014; Frerigmann *et al.*, 2014; Aarabi *et al.*, 2016, 2020) and in seed storage protein regulation (Aarabi *et al.*, 2021). In the Plant Regulomics database two new MYB TFs have been identified (Table 3) which deserve further analysis. Hitherto, MYB3 (AT1G22640) has been assigned to phenylpropanoid metabolism (Kim *et al.*, 2022) but also to plant growth control through phytosulfokines, sulfur compounds modulating auxin responses (Badola *et al.*, 2022). Phytosulfokines, which can be viewed as peptide hormones, are insufficiently studied in terms of their relationship to sulfate deprivation or OAS signaling (Komori *et al.*, 2009; Kopriva *et al.*, 2012). Under stress conditions, whether nutrient deprivation or other stresses involving ROS, phenylpropanoid biosynthesis is induced to alleviate stress symptoms. In the context of sulfate deprivation, MYB TFs have been associated with this response, among them *PAP1/MYB75* (AT1G56650), which controls anthocyanin biosynthesis (Nikiforova *et al.*, 2005a; Wulff-Zotele *et al.*, 2010; Watanabe and Hoefgen, 2019). Interestingly, the dataset (GSE157765) of Dietzen *et al.* (2020) indicates jointly coordinated repression of MYB75 by EIL3/SLIM1 and EIL1, which requires an induction independent of the EIL3/SLIM1 regulatory circuit. Furthermore, in addition to MYB3, the TFs HY5 and PIF3, which regulate anthocyanin accumulation and phenyl-propanoid biosynthesis (Oyama *et al.*, 1997; Kim *et al*., 2003; Shin *et al.*, 2007; Kim *et al*., 2022), seem to be involved in the regulation of the OAS-responsive gene network (Table 3). For the second MYB-related TF (AT3G10580), scarce information is available to date, but as it targets five genes of the OAS cluster expression network, its future analysis is recommended. AT3G10580 has been identified to potentially interact with the above-mentioned MYB3 which controls anthocyanin and lignin biosynthesis under salt stress (Kim *et al.*, 2022).

An additional level of regulation during sulfur deprivation is provided through epigenetic modifications associated with the OAS cluster gene *MSA1* (Hubberten *et al.*, 2012b) that is involved in DNA and other regulatory methylation reactions (Huang *et al.*, 2016). Further, NRPE1 is a TF involved in RNA-directed DNA methylation presumably playing a role in gene control, seed development, and pathogen responses (Sasaki *et al.*, 2019; Miao *et al.*, 2021; Wang *et al.*, 2021). NRPE1 binds to the promoters of six genes of the co-expression network (Table 3). Its relationship to the OAS signaling pathway or sulfate availability is unclear and needs further investigations.

Among the identified TFs are several whose link to OAS signaling and/or sulfate metabolism is still unclear. NFYB2, NFYC2, and PIF4 (Table 3) are all known to be involved in regulating genes responding to nutrient levels (Brumbarova and Ivanov, 2019). Moreover, there is a connection between sulfate and ABA since reduced sulfate availability results in reduced ABA content in the plant tissue (Cao *et al.*, 2014). This explains the presence of three TFs, DTAF, HB6, and HB7 (Ré *et al.*, 2014; Gaudinier *et al.*, 2018; Ma *et al.*, 2021) (Table 3). Moreover, TFs involved in the circadian clock and response to light were revealed. OAS has been indicated to accumulate in the middle of the night-time (Espinoza *et al.*, 2010) or immediately after the plants are transferred from light into the dark (Caldana *et al.*, 2011). OAS accumulation results in the regulation of numerous genes, such as the core OAS cluster genes (Hubberten *et al.*, 2012b). TFs such as RVE8, PIF3, LHY, and CCA1 (Pérez-García *et al.*, 2015; Wang *et al.*, 2021; Hao *et al.*, 2022) which regulate genes involved in light responses, might potentially regulate a significant number of genes from the OAS co-expression network (Table 3).

## Conclusion

OAS has long been considered a signal within the sulfate deprivation response (Saito, 2000; Hirai *et al.*, 2003) with increasing evidence in recent years (Hubberten *et al.*, 2012b; Aarabi *et al.*, 2016, 2020). OAS, though an inherent precursor of cysteine synthesis, has been shown to accumulate not only under low sulfur conditions (Maruyama-Nakashita *et al.*, 2006; Hubberten *et al.*, 2012a), but also under different stresses. These stresses, such as heavy metal exposure (Jalmi *et al.*, 2018), ROS-inducing herbicide treatment (Lehmann *et al.*, 2009, 2012), jasmonate accumulation (Jost *et al.*, 2005), or shifts from light to darkness (Caldana *et al.*, 2011) provoke ROS accumulation. Notably, sulfate availability is not altered under these conditions. Experimentally this has been corroborated by overproducing SERAT leading to OAS accumulation (Hubberten *et al.*, 2012b). All these conditions lead to the induction of a core set of genes, the OAS cluster genes.

All OAS cluster core genes, except APR3, seem to be regulated, at least in response to sulfate deprivation, by the commonly accepted central regulator of plant sulfate metabolism, SLIM1/EIL3 (Tables 2, 3). *SDI2* seems rather to be repressed by SLIM1 while *SDI1* needs the presence of SLIM1 under sulfur-depleted conditions (Dietzen *et al.*, 2020). The promoters of the SLIM1/EIL3-regulated genes contain the known *cis*-elements UPE-box and TEBs (Wawrzyńska *et al.*, 2010, 2022), and SURE elements were recently proven to bind EIL2/SLIM1(Rakpenthai *et al.*, 2022). *In silico* and *in vitro* analyses of the promoter regions revealed many more putative *cis*-elements, indicating that further TFs are potentially able to bind to the promoter regions (Table 3). This, together with the fact that *APR* is an OAS cluster gene but not subject to SLIM1 control, suggests that other regulatory circuits control the expression of the OAS cluster genes in response to OAS rather than SLIM1 alone. From the OAS co-expression cluster, *BGLU28*, *BGLU30*, and *PYD* also display features indicating independent sulfate deprivation and OAS signaling pathways, probably acting in parallel under sulfate-deprived growth conditions. Having a knockout SLIM1 line available (Wawrzyńska *et al.*, 2022) now provides the possibility to test this experimentally. Based on the fact that stresses other than sulfate deprivation result in OAS accumulation and the common responses of the OAS cluster genes, we have to assume that OAS signaling is distinct from sulfate deprivation signaling, though both coincide under sulfate-deprived growth conditions. This decoupling of the OAS and the sulfur deprivation response is supported by the fact that in potato (*Solanum tuberosum*) several sulfate deprivation responses such as SULTR expression and increased sulfate uptake capacity precede OAS accumulation (Hopkins *et al.*, 2005).

Further evidence is provided by the extended OAS cluster co-expression network (Fig. 2; Table 1) as these genes are co-expressed over a wide range of conditions but are only partially sulfate deprivation-responsive genes or induced by OAS. Hence, we have to conclude the existence of an additional regulatory pathway specific for distinct stresses and, positive as well as negative, interference of the TF by modulating their target genes in response to various stresses and signals. Such stress-specific response pathways are also indicated through the differential inducibility of the OAS-synthesizing SERAT genes. Moreover, the fact that some genes, such as *APR* or *LSU* genes, are up-regulated in sulfur deprivation conditions (Howarth *et al.*, 2003; Dietzen *et al.*, 2020), but not or weakly upon OAS treatment (Hubberten *et al.*, 2012b), indicates that there are further regulatory factors affecting gene expression, other than just the signal molecule OAS, to be considered.

SDIs are not TFs but interact with them and regulate gene expression of, for example, MYB28 (Aarabi *et al.*, 2016, 2020) or regulate the accumulation of sulfur-rich seed storage proteins in seeds (Aarabi *et al.*, 2021). The latter might be the original function present in all plants, while GSL regulation is a *Brassicaceae*-specific acquisition. In addition, MSA1 and potentially NRPE1 through their methylation activity are non-TF regulators epigenetically affecting sulfur homeostasis (Huang *et al.*, 2016), and perhaps also other processes. This leads to the conclusion that in addition to TFs, other regulators will have to be considered as regulating either the sulfate deprivation response or other, OAS-specific stress responses. In this context, while *miRNA395* has been shown to affect SULTR activity and sulfate allocation under sulfate deprivation in Arabidopsis (Davidian and Kopriva, 2010), other RNA-based or proteinaceous regulators still need to be considered. To further understand the regulation of the OAS cluster genes and, hence, their downstream effects on sulfate metabolism or other metabolic pathways, the positioning and structure of *cis*-elements within various promoters will need more attention. There are sets of TFs targeting similar *cis*-elements or overlapping *cis*-elements for different TFs (Rakpenthai *et al.*, 2022). What determines the priority of binding? Does binding alter existing promotor features such as palindromic structures? Could this lead to acceptance or exclusion of TFs targeting OAS cluster gene promoters?

Eventually, neither OAS signal perception nor the signal transduction pathways are reliably resolved yet.

## Data Availability

Raw data that support the findings of this study, such as ATTED results, are available from the corresponding author, upon request. Further data which were used to generate the heatmap in Fig. 1, can be found from the datasets mentioned in Fig. 1B.

## Abbreviations

ABA: abscisic acid
APK: APS kinase
APR: APS reductase
APS: adenosine 5ʹ-phosphosulfate
CSC: cysteine synthase complex
DAP-seq: DNA Affinity Purification and sequencing
ER: endoplasmic reticulum
GO: Gene Ontology
GSL: glucosinolate
GSH: glutathione
MeJa: methyljasmonate;
OAS: *O*-acetyl-serine
OASTL: *O*-acetylserine-thiol-lyase
ROS: reactive oxygen species
SERAT: serine acetyltransferase
TF: transcription factor
